# A Three-Dimensional Cell Culture System To Model RNA Virus Infections at the Blood-Brain Barrier

**DOI:** 10.1128/mSphere.00206-17

**Published:** 2017-06-21

**Authors:** John C. Bramley, Coyne G. Drummond, Nicholas J. Lennemann, Charles A. Good, Kwang Sik Kim, Carolyn B. Coyne

**Affiliations:** a Department of Microbiology and Molecular Genetics, University of Pittsburgh, Pittsburgh, Pennsylvania, USA; b Department of Pediatrics, University of Pittsburgh, Pittsburgh, Pennsylvania, USA; c Center for Microbial Pathogenesis, Children’s Hospital of Pittsburgh of UPMC, Pittsburgh, Pennsylvania, USA; d Division of Infectious Diseases, The Johns Hopkins University, Baltimore, Maryland, USA; UT Southwestern Medical Center

**Keywords:** blood-brain barrier, coxsackievirus, dengue virus, echovirus, tight junction, Zika virus, enterovirus, flavivirus

## Abstract

The blood-brain barrier (BBB) comprises the foremost protective barrier in the brain and is composed in part of a layer of microvascular endothelial cells that line the capillaries surrounding the brain. Here, we describe a human three-dimensional (3-D) cell-based model of the BBB microvascular endothelium that recapitulates properties of these cells *in vivo*, including physiologically relevant transcriptional profiles, the capacity to induce potent antimicrobial innate immune signaling, and the ability to resist infection by diverse RNA viruses, including members of the enterovirus (coxsackievirus B, echovirus 11, enterovirus 71, poliovirus) and flavivirus (dengue virus, Zika virus [ZIKV]) families. We show that disruption of apical tight junctions by proinflammatory cytokine tumor necrosis factor alpha (TNF-α) sensitizes 3-D-cultured BBB cells to ZIKV infection and that 3-D derived BBB cells can be used to model the transmigration of ZIKV-infected monocytes across the endothelial barrier to access underlying astrocytes. Taken together, our findings show that human BBB microvascular endothelial cells cultured in 3-D can be used to model the mechanisms by which RNA viruses access the central nervous system (CNS), which could be used for the development and screening of therapeutics to limit this event.

**IMPORTANCE** Neurotropic viral infections are significant sources of global morbidity and mortality. The blood-brain barrier (BBB) is composed in part of a layer of microvascular endothelial cells and functions to restrict viral access to the brain. *In vitro* models that recapitulate many of the properties of the human BBB endothelium are lacking, particularly with respect to the unique cellular and immunological mechanisms by which these cells restrict viral infections of the brain. Here, we developed a three-dimensional cell culture model that recapitulates many of the morphological and functional properties of the BBB microvasculature and apply this model to the study of RNA virus infections. The model we describe can therefore be used to study a variety of aspects of BBB physiology, including the mechanisms by which viruses might access the CNS, and could be used for the development and screening of antiviral therapeutics to limit this important step in viral pathogenesis.

## Abstract (precis)

Neurotropic viral infections are significant sources of global morbidity and mortality. The blood-brain barrier (BBB) is composed in part of a layer of microvascular endothelial cells and functions to restrict viral access to the brain. *In vitro* models that recapitulate many of the properties of the human BBB endothelium are lacking, particularly with respect to the unique cellular and immunological mechanisms by which these cells restrict viral infections of the brain. Here, we developed a three-dimensional cell culture model that recapitulates many of the morphological and functional properties of the BBB microvasculature and apply this model to the study of RNA virus infections. The model we describe can therefore be used to study a variety of aspects of BBB physiology, including the mechanisms by which viruses might access the CNS, and could be used for the development and screening of antiviral therapeutics to limit this important step in viral pathogenesis.

## INTRODUCTION

Neurotropic viruses are significant sources of human disease and are associated with global morbidity and mortality (reviewed in reference 1). The blood-brain barrier (BBB) comprises the foremost protective barrier in the brain and functions to prevent the free passage of viruses from the bloodstream into the central nervous system (CNS). The BBB is composed in part from a layer of polarized microvascular endothelial cells (MEC) lining the capillaries surrounding the brain and functions to restrict viral access into the CNS, largely owing to the presence of tight junctions (TJs) that limit the free flow of ions, solutes, and macromolecules across the endothelium. However, the BBB endothelium is not simply a passive barrier but also plays an active role in communicating signals between the systemic circulation and the brain as well as actively participating in the immunological defense of the CNS.

Neurological infections caused by RNA viruses can be associated with encephalitis, meningitis, paralysis, or even death. Viral access to the CNS via the BBB can occur through several routes, which include direct infection of the endothelium, passage across the paracellular space following junctional dysregulation (often as a result of cytokine-induced damage), and transmigration of infected immune cells across the endothelium (reviewed in reference 2). Despite the importance of the BBB in protecting the CNS from viruses, *in vitro* models that fully recapitulate many of the key properties of the human BBB endothelium are lacking, particularly with respect to the unique cellular and immunological mechanisms by which these cells restrict viral infections. For example, a limitation of existing cell line-based models of BBB endothelial cells is their failure to inhibit solute and ion flow with the same efficiency as their *in vivo* counterparts, as evidenced by their low resistance to the flow of ions and solutes across the endothelium (3–6). In addition to differences in barrier function, many *in vitro* models of the BBB endothelium also fail to recapitulate the unique transcriptional profile of these cells, which includes expression of BBB-specific transporters, cytoskeletal factors, and TJ-associated components, among others (7).

Human-based cell models of the BBB microvasculature include those using immortalized cell lines (8–10) or human pluripotent stem cells that can be differentiated into microvascular endothelial cells (11). However, many existing models of the BBB fail to incorporate shear stress into the culture system, to which the apical surfaces of the BBB endothelium is constantly exposed *in vivo*. The importance of the effect of shear forces on the BBB is clear—cultured microvascular endothelial cells respond to shear stress by inhibiting cellular proliferation, increasing junctional barrier and cytoskeletal functioning, and enhancing the capacity to interact with immune cells (12–14). The limitations of existing BBB models have led to the development of systems that incorporate BBB microvascular cells, cultured either monotypically or with other relevant cells types such as astrocytes, and shear stress, represented by either hollow fibers or microfluidic “chip” technologies that impart shear stress via the flow of culture medium across the surfaces of the endothelium (reviewed in reference 15). While each of these systems has distinct advantages and disadvantages, many are difficult to apply to the study of infectious agents, given the need to use perfusion systems with potentially large volumes of virus-filled medium and/or the small biomass resulting from the use of very low cell numbers.

Given the limitations of existing BBB models, we sought to develop a three-dimensional (3-D) cell culture model of human BBB microvascular endothelial cells and to apply this model to the study of RNA viruses. We used human brain microvascular endothelial cells (HBMEC) (9), an immortalized human BBB microvascular endothelial cell line, grown in a rotating wall vessel (RWV) bioreactor, which recapitulates the quiescent microgravity environment and the exposure of cells to shear (16). Using this system, we found that culturing of HBMEC in 3-D resulted in physiologically relevant transcriptional changes, induced their capacity to potently mount antimicrobial innate immune defenses, and conferred high resistance to infection by diverse RNA viruses, including members of the enterovirus and flavivirus families. Finally, we found that disruption of endothelial tight junctions (TJs) by treatment with EDTA or the proinflammatory cytokine tumor necrosis factor-α (TNF-α) sensitized 3-D-cultured BBB cells to RNA virus infections. Finally, we show that 3-D-derived HBMEC can be used to model monocyte-mediated transmigration across the endothelium. Taken together, our findings show that 3-D culturing of BBB microvascular endothelial cells using an RWV bioreactor can be used to model a variety of aspects of BBB physiology, including the mechanisms by which viruses might access the CNS, and could be used for the development and screening of antiviral therapeutics to limit this important step in viral pathogenesis.

## RESULTS

### Establishment of 3-D HBMEC cell cultures.

**FIG 1:**
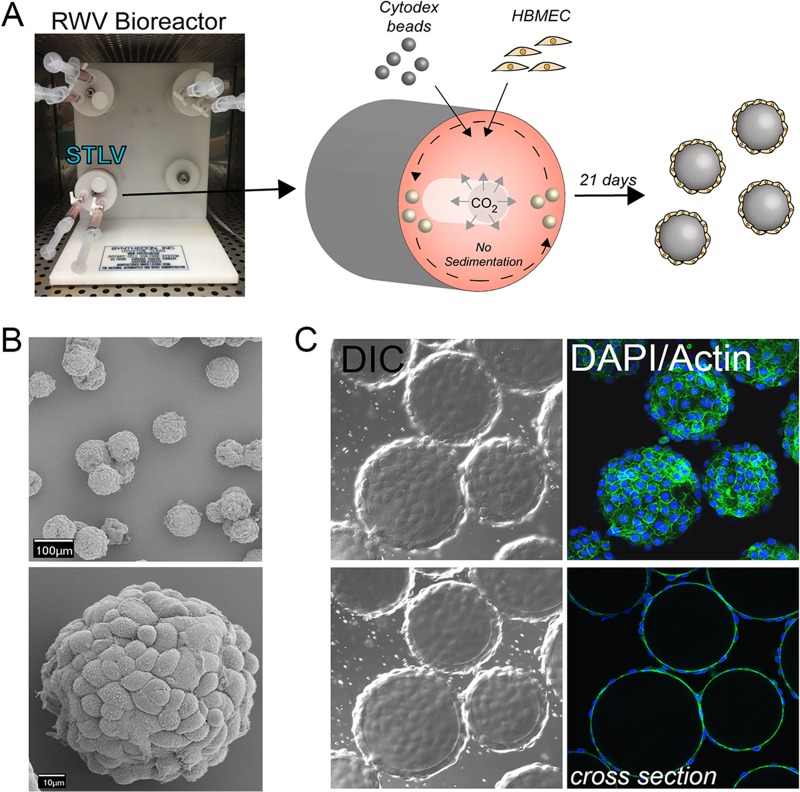
RWV bioreactor culturing of HBMEC under 3-D conditions. (A) Schematic of the RWV bioreactor. Slow-turning lateral vessels (STLV) attached to the RWV bioreactor were filled with tissue culture medium and cytodex beads coated with HBMEC for 21 days. Following this culture period, cell-covered beads were removed from the STLVs for all subsequent downstream studies. (B) Scanning electron micrographs (SEM) of HBMEC-coated beads following 21 days of culture. (C) Confocal micrographs for actin (green) in HBMEC cultured for 21 days. Differential interference contrast (DIC) data are shown at left, and DAPI-stained nuclei are shown in blue. The bottom row shows a cross-section through beads.

The RWV bioreactor is based on the attachment of slow-turning lateral vessels (STLVs), which are filled with cell culture medium and contain cells attached to porous, extracellular matrix (ECM)-coated beads (although other scaffolds can be used in the system) (16) (schematic, Fig. 1A), to a rotating base. We established this system for HBMEC using Cytodex-3 collagen-coated porous dextran beads and cultured cells for 21 days prior to removal from STLVs, at which time the cell-coated beads were used for subsequent downstream applications (schematic, Fig. 1A). We found that HBMEC were amenable to monotypic culturing within the STLV and that these cells fully coated the beads during the culture period to form a uniform and single layer of cells as assessed by scanning electron microscopy (SEM) and actin localization (Fig. 1B and C).

### Transcriptional profiling of HBMEC cultured under 3-D conditions.

**FIG 2:**
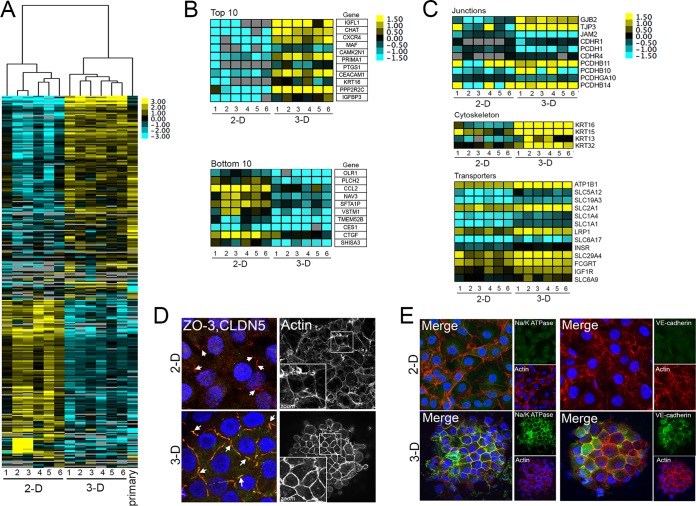
Transcriptional profiling of HBMEC grown under 3-D conditions. (A) Hierarchical-clustering heat map of genes differentially expressed (*P* < 0.001) between six independent cultures of HBMEC grown under 2-D or 3-D conditions or primary HBMEC based on log(RPKM) values. Primary HBMEC were used at approximately passage 8 postisolation and were cultured under “2-D” conditions for 48 h prior to RNA isolation. (B) Heat maps of the top 10 (top) or bottom 10 (bottom) differentially expressed transcripts between 2-D- and 3-D-cultured HBMEC. At right, a table containing gene names is shown. (C) Heat maps of transcripts associated with specific BBB-associated processes such as junctions (top), cytoskeleton (middle), and transporters (bottom) between 2-D- and 3-D-cultured HBMEC. Gene names are indicated at right. (D and E) Confocal micrographs for ZO-3 (red) and claudin-5 (green) or actin (white at right) (D) or Na/K ATPase (green, left panel) or VE-cadherin (green, right panel) and actin (red in both panels) (E) in 2-D- or 3-D-cultured HBMEC. The color intensity in panels A to C indicates the level of gene expression (yellow for upregulation and blue for downregulation), and gray indicates that no reads were detected for that transcript.

To explore the differences between HBMEC cultured in two dimensions (2-D) and those cultured in 3-D, we conducted whole transcriptome sequencing (RNAseq) analyses of six independent 2-D or 3-D preparations and identified differentially expressed genes using the DESeq2 package in R (17). For comparisons of cells cultured in 2-D, cells were grown on either collagen-coated Transwell inserts (samples 1 and 2) or tissue culture-coated plastic plates (samples 3 to 6) for a period of 2 to 7 days. We identified 3,904 differentially expressed transcripts (coding and noncoding) in HBMEC cultured in 2-D versus 3-D (*P* < 0.001) (Fig. 2A). Gene set enrichment analysis (GSEA) (18) revealed the upregulation of several pathways, including metabolic pathways (false-discovery rate [FDR] = 0.002) and those associated with the development of apical junctions (FDR = 0.038) (see Fig. S1A in the supplemental material), among others. The most potently downregulated pathways were involved in the regulation of the cell cycle (including E2F targets [FDR = 0.000] and G2M checkpoints [FDR = 0.000]) and in cell growth and division (c-Myc targets [FDR = 0.000]) (Fig. S1A). Interestingly, we also found that 640 transcripts differentially expressed between 2-D and 3-D cultures of HBMEC were also differentially expressed (*P* < 0.05) when another BBB microvascular endothelial cell line, the hCMEC/D3 cell line (10), was cultured in 3-D (see Table S1 in the supplemental material; see also Fig. S1B and C). To determine if any of the transcripts differentially expressed by the culturing of HBMEC in 3-D were shared with primary BBB endothelial cells, we conducted RNAseq analyses using primary HBMEC and found by hierarchical clustering that 3-D-cultured HBMEC and primary HBMEC transcriptional changes clustered together (top brackets, Fig. 2A). Moreover, differential expression analyses revealed that all of the differentially expressed transcripts in 2-D-cultured and primary HBMEC (*n* = 230; *P* < 0.001) were also differentially expressed in 2-D- and 3-D-cultured HBMEC (Fig. S1D).

10.1128/mSphere.00206-17.1 FIG S1  (A) Gene set enrichment plots for pathways up- or downregulated following culturing of HBMEC under 3-D conditions. (B) Scanning electron micrograph of hCMEC/D3 cells grown for 21 days in the RWV bioreactor. (C) Hierarchical-clustering heat map of genes differentially expressed (*P* < 0.001) between two independent cultures of hCMEC/D3 grown under 2-D conditions or 3-D conditions based on log(RPKM) values. The color intensity indicates the level of gene expression (yellow for upregulation and blue for downregulation), and gray indicates that no reads were detected for that transcript. (D) Venn diagram denoting the overlap of transcripts differentially expressed between 2-D- and 3-D-cultured HBMEC (purple), 2-D and primary HBMEC (blue), and 3-D and primary HBMEC (green). (E) RT-qPCR for analysis of CHAT or H19 in 3-D-cultured HBMEC for the indicated days. (F) RT-qPCR for the indicated transcripts in 2-D (gray) or 3-D (red) cultures of HBMEC, or in HBMEC cultured on Cytodex-3 beads for 7 days (green). (G) RT-qPCR for the indicated transcripts in 2-D- or 3-D-cultured HBMEC. Data are representative of results from six independent STLVs. Data in panel E to G are shown as means ± standard deviations (*, *P* < 0.05; **, *P* < 0.01; ***, *P* < 0.001). Download FIG S1, PDF file, 3 MB. Copyright © 2017 Bramley et al. 2017 Bramley et al. This content is distributed under the terms of the Creative Commons Attribution 4.0 International license.

10.1128/mSphere.00206-17.6 TABLE S1  Lists of genes whose differential expression between 2-D- and 3-D-cultured HBMEC and hCMEC/D3 cells are shared. Gene names and log_2_(fold change) values as determined by the DeSeq2 package in R are shown. Upregulated genes are shown in green, and downregulated genes are shown in red. Download TABLE S1, XLSX file, 0.2 MB. Copyright © 2017 Bramley et al. 2017 Bramley et al. This content is distributed under the terms of the Creative Commons Attribution 4.0 International license.

The most highly differentially expressed genes between 2-D and 3-D cultures of HBMEC included those encoding choline *O*-acetyltransferase (CHAT), an enzyme involved in the synthesis of acetylcholine; C-X-C chemokine receptor type 4 (CXCR4), which is expressed at the BBB (19); calcium/calmodulin-dependent protein kinase II inhibitor 1 (CAMK2N1), which controls the cell cycle and is specifically expressed in the brain (20); and protein phosphatase 2 regulatory subunit B gamma (PPP2R2C), brain-specific protein phosphatase 2A (21), among others (Fig. 2B, top). Results from RNAseq studies were confirmed by reverse transcription-quantitative PCR (RT-qPCR) (Fig. S1E). Conversely, the most extensively downregulated genes included those encoding connective tissue growth factor (CTGF), also known as connective tissue growth factor (CCN2), a major platelet-derived growth factor-related mitogen secreted by human vascular endothelial cells (22), and neuron navigator 3 (NAV3), which is expressed in the developing brain (23) (Fig. 2B, bottom). We found that the level of induction of select transcripts correlated with the number of days in culture, with many transcriptional changes induced within 5 to 10 days of initiating the 3-D culture (Fig. S1E). Importantly, we also found that the culturing of HBMEC on cytodex beads under static conditions did not induce transcriptional changes of the kind seen with cells grown using the RWV bioreactor (Fig. S1F, “2D^beads^,” green), supporting the hypothesis that the exposure of cells to the shear forces in the STLV, and not the culturing of cells on cytodex beads alone, induces the observed transcriptional changes.

To identify whether culturing of HBMEC in 3-D altered specific transcripts associated with the functional properties of the BBB, we analyzed the RNAseq transcriptional data for genes specifically expressed in the BBB using previously published data sets (7, 24–26). These included genes associated with junctional complexes, the actin cytoskeleton, and BBB-enriched transporters. In many cases, we found a significant upregulation of transcripts associated with a BBB phenotype in 3-D cultures, including those encoding junction-associated proteins such as connexin-26 (GJB2); a gap junction component, ZO-3 (TJP3); and a number of cadherins and protocadherins, which maintain the adherens junctions (Fig. 2C, top). In addition, a number of cytoskeleton-associated components, including several members of the cytokeratin family, were upregulated in cells grown in 3-D (Fig. 2C, middle). Finally, we observed the upregulation of a number of cell surface transporters known to be enriched in the BBB, such as low-density lipoprotein receptor-related protein 1 (LRP1), a key BBB-expressed protein and peptide transporter (27); glucose transporter SLC2A1 (Glut-1); and others (Fig. 2C, bottom). These results were confirmed by RT-qPCR (Fig. S1G).

To further confirm our RNAseq findings, we next investigated the cellular distribution of molecules and pathways enriched by culturing of HBMEC under 3-D conditions. Consistent with our transcriptional studies, we found that HBMEC cultured in 3-D exhibited well-formed TJs compared to 2-D-cultured cells, as shown by an enrichment of actin at the junctional complexes and enhanced expression of both ZO-3 and claudin-5 (Fig. 2D). Moreover, we found that 3-D-cultured HBMEC displayed substantial enhancement in the expression of sodium-potassium ATPase subunit alpha 1 (Na^+^/K^+^ ATPase-1) and that this transporter was localized to the junctional complexes based upon colocalization with actin (Fig. 2E, left panels). In addition, we noted increased levels of vascular endothelial cadherin (VE-cadherin), which was enriched at the junctions of cells grown in 3-D (Fig. 2E, right panels), and of claudin-1, a TJ-associated component (Fig. S1G). Collectively, these data show that culturing of HBMEC in 3-D in the RWV bioreactor elicits physiologically relevant transcriptional changes.

### HBMEC removed from 3-D cultured cytodex beads retain a more BBB-like phenotype.

**FIG 3:**
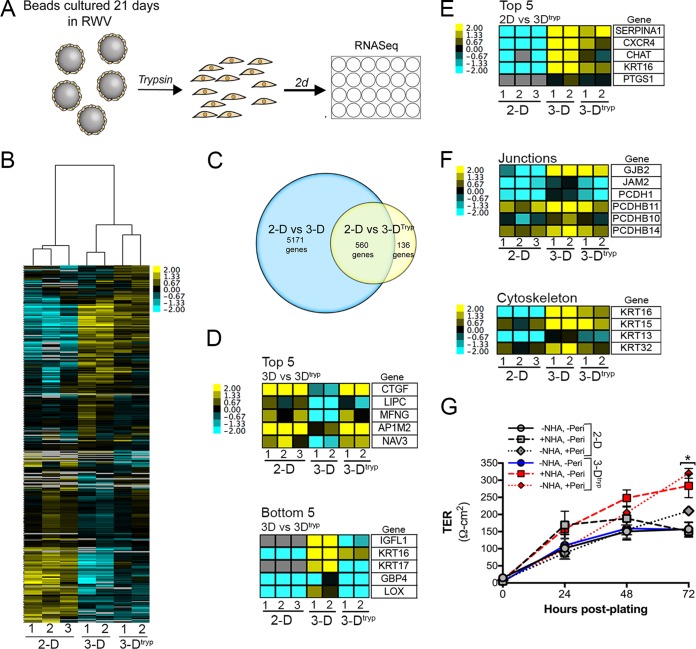
Isolated 3-D-cultured HBMEC grown under 2-D conditions retain many BBB-like properties. (A) Schematic for the trypsin-mediated removal of 3-D-grown HBMEC (3-D^tryp^). HBMEC are cultured on the RWV bioreactor for 21 days, and cells are removed by trypsin-mediated digestion. Isolated cells are then plated for 2 to 3 days in 24-well tissue culture plates for RNAseq analyses or are cocultured with primary human astrocytes or primary pericytes in Transwell inserts for measurements of transendothelial electrical resistance (TER). (B) Hierarchical-clustering heat map of genes differentially expressed (*P* < 0.001) between three independent cultures of HBMEC grown under 2-D conditions and two independent cultures of matched 3-D or 3-D^tryp^ cells based on log(RPKM) values. (C) Venn diagram of the overlap of transcripts differentially expressed between 2-D- and 3-D-cultured HBMEC (blue) and 2-D and 3-D^tryp^ cultures (yellow). (D) Heat maps of transcripts associated with the top 5 (top row) or bottom 5 (bottom row) most highly differentially expressed genes between 3-D and 3-D^tryp^ cultures. At right, a table containing gene names is presented. (E) Heat map of the top 5 most highly differentially expressed transcripts between 2-D and 3-D^tryp^ cultures, highlighting the overlap of 3-D-cultured HBMEC. At right, a table containing gene names is presented. (F) Heat maps of transcripts associated with specific BBB-associated processes such as junctions (top) and cytoskeleton (bottom) between 2-D and 3-D^tryp^ cultures, highlighting the overlap of 3-D-cultured HBMEC. At right, a table containing gene names is presented. (G) TER values (expressed in ohms per square centimeter) in monotypic (no primary normal human astrocytes [NHA] or pericytes) cultures of 2-D (gray, solid line) or 3-D^tryp^ (blue, solid line) HBMEC at the indicated time (in hours) postplating (solid lines) or in 2-D (gray, dashed lines) or 3-D^tryp^ (red, dashed lines) HBMEC cocultured with NHA or pericytes as shown in the schematic in panel A (hatched lines). Data are shown as means ± standard deviations and are representative of results of eight technical replicates from two independent STLV cultures grown independently (*, *P* < 0.05). The color intensity in panels B and D to F indicates the level of gene expression (yellow for upregulation and blue for downregulation), and gray indicates that no reads were detected for that transcript.

An inherent limitation of the culturing of HBMEC in the RWV bioreactor is the need to grow cells on cytodex beads, which limits the ability to easily perform assays such as measuring transendothelial resistances (TER) and coculturing cells with human astrocytes or pericytes, which both play roles in the development and function of the BBB (28–30). To overcome these limitations, we optimized the trypsin-mediated removal of HBMEC from cytodex beads following their culturing on the RWV bioreactor and determined whether this removal influenced their transcriptional profile by RNAseq or their junctional integrity (schematic, Fig. 3A). We found that cells removed from beads after 21 days in 3-D culture (3-D^tryp^) were viable as assessed by trypan blue staining and exhibited an intact actin cytoskeleton following their removal and subsequent plating (Fig. S2A). In addition, the morphology of 3-D^tryp^ cells was similar to that seen with 2-D-cultured HBMEC, as assessed by bright-field microscopy (Fig. S2B).

10.1128/mSphere.00206-17.2 FIG S2  (A) Confocal micrographs for actin (green) in 3-D or 3-D^tryp^ cells (isolated from cells grown in the same STLV) 24 h following removal. (B) Bright-field microscopy images of 2-D HBMEC or two independent preparations of 3-D^tryp^ cells. (C) Schematic of the Transwell system established for the coculturing of 2-D- or 3-D-derived HBMEC and primary human pericytes or astrocytes. At right top, a confocal micrograph cross-section is shown of HBMEC on the apical side of the Transwell membrane and primary human pericytes on the basolateral side of the Transwell stained with actin (in red). At right bottom, primary human astrocytes plated in the basolateral chamber were immunostained for GFAP (green). In both panels, DAPI-stained nuclei are shown in blue. Download FIG S2, PDF file, 2.7 MB. Copyright © 2017 Bramley et al. 2017 Bramley et al. This content is distributed under the terms of the Creative Commons Attribution 4.0 International license.

Using RNAseq, we defined the transcriptional changes induced by the removal of HBMEC from beads and their subsequent growth for ~48 h on tissue culture-treated plates (Fig. 3B). We found that ~80% of the transcripts differentially expressed between 2-D- and 3-D^tryp^-cultured HBMEC were differentially expressed between 2-D- and 3-D-cultured cells (Fig. 3C). However, several of the genes that were similarly expressed between 3-D^tryp^- and 2-D-cultured cells, such as those encoding CTGF, NAV3, insulin-like growth factor-like family member 1 (IGFL1), and select cytokeratins (KRTs), were those most highly differentially expressed in cells grown in 3-D, suggesting that removal from the STLV induced rapid changes in the expression of select transcripts, likely through alterations in cellular junctions and cytoskeleton by trypsin-mediated digestion (Fig. 3D). Importantly, the differential expression of many transcripts was maintained in 3-D^tryp^ cells relative to cells cultured in 2-D, including the expression of CXCR4, CHAT, and junction- and cytoskeleton-associated components (Fig. 3E and F). Collectively, these data suggest that many transcripts that were altered by culturing of cells in the RWV bioreactor, and which are associated with a functional BBB, were maintained for at least several days following their trypsin-mediated removal from cytodex beads and subsequent growth in 2-D.

Cultured BBB cell lines exhibit low TER values, indicating that BBB microvascular endothelial cells cultured under standard conditions do not recapitulate the barrier function of these cells *in vivo* (3–6). Because we found that the trypsin-mediated removal of HBMEC following their culturing in 3-D resulted in the retention of the overall transcriptional signature of cells isolated immediately following their removal from STLVs, we next assessed whether these cells exhibited differences in junctional barrier properties. We found that monotypic cultures of HBMEC grown under 2-D conditions and of those isolated following 3-D culturing (3-D^tryp^) yielded similar TER values (Fig. 3G). However, given that the coculturing of BBB microvascular endothelial cells with human astrocytes promotes the development of barrier function (reviewed in reference 30) and that pericytes play a role in the formation and maintenance of the BBB (28, 29), we established cocultures of primary normal human astrocytes (NHA) or primary human pericytes and either 2-D or 3-D^tryp^ HBMEC cultured in Transwell inserts (Fig. 3A; Fig. S2C). Under all conditions, TER values increased during the initial 24 h postculturing (Fig. 3G). However, we found that, whereas the TER values of 2-D-derived HBMEC plateaued between 24 and 48 h postplating and then decreased by 72 h postplating, the Ter values of 3-D^tryp^ HBMEC cocultured with NHA or pericytes continued to increase until 72 h postplating to values that were significantly higher than those observed in 2-D-cultured cells (Fig. 3G).

### HBMEC cultured in 3-D induce antimicrobial innate immune signaling.

**FIG 4:**
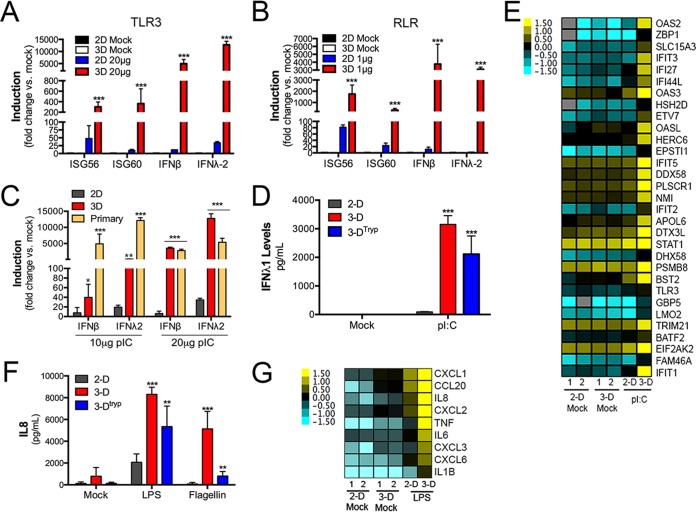
Enhanced innate immune signaling in 3-D-cultured HBMEC. (A and B) RT-qPCR for ISG56, ISG60, IFN-β, or IFN-λ2 in 2-D- or 3-D-cultured HBMEC exposed to 20 μg of poly(I·C) to stimulate TLR3 (A) or transfected with 1 μg of poly(I·C) to activate RLRs (B). (C) RT-qPCR for IFN-β or IFN-λ2 from 2-D- or 3-D-cultured HBMEC or from primary HBMEC exposed to 10 μg or 20 μg poly(I·C). (D) ELISA for IFN-λ1 (with results expressed in picograms per milliliter) in 2-D- or 3-D-cultured HBMEC or in 3-D^tryp^ cultures exposed to 20 μg of poly(I·C). In the experiments whose results are presented in panels A to D, cells were exposed to the indicated ligands for ~24 h and were exposed after 2 days postplating for 2-D or 3-D^tryp^ cells. (E) Heat map of known ISGs in mock-treated 2-D- or 3-D-cultured HBMEC or in cultures exposed to 10 μg of poly(I·C) for ~24 h based upon log(RPKM) values as assessed by RNAseq. (F) ELISA for IL-8 (with results expressed in picograms per milliliter) in 2-D- or 3-D-cultured HBMEC or in 3-D^tryp^ cultures exposed to LPS or flagellin. (G) Heat map of proinflammatory transcripts in mock-treated 2-D- or 3-D-cultured HBMEC or in cultures exposed to 500 ng LPS based upon log(RPKM) values as assessed by RNAseq. Data in panels A to D, F, and G represent fold changes from mock-treated control results and are shown as means ± standard deviations (*, *P* < 0.05; **, *P* < 0.01; ***, *P* < 0.001). Data are representative of cells isolated from at least four independent STLV cultures, with experiments performed in duplicate or triplicate for all cultures. The color intensity in panels E and G indicates the level of gene expression (yellow for upregulation and blue for downregulation), and gray indicates that no reads were detected for that transcript.

In addition to its physical barrier function, the BBB microvascular endothelium acts as a key immunological barrier. Given the requirement to potently induce antimicrobial innate immune signals in response to diverse microbes, we next determined whether the culturing of HBMEC in 3-D altered the sensing of microbes or their propagation of antimicrobial signals. To do this, we used synthetic ligands of RNA viruses {polyriboinosinic:polyribocytidylic acid [poly(I·C)]} and bacteria (lipopolysaccharide [LPS] and flagellin), which are detected by Toll-like receptor 3 (TLR3), poly(I·C), TLR4 (LPS), or TLR5 (flagellin). We found that 3-D cultures of HBMEC responded more efficiently to poly(I·C) (either used in the medium to stimulate TLR3 signaling or transfected to induce RIG-I-like receptor [RLR] signaling) than cells cultured in 2-D, as assessed by RT-qPCR for interferon-stimulated genes (ISGs) and both type I (beta) interferon (IFN-β) and type III (lambda) interferon (IFN-λ2) (Fig. 4A and B; see also Fig. S3A). Interestingly, we also found that primary HBMEC responded to poly(I·C) stimulation more robustly than 2-D-cultured cells, with levels similar to those observed in 3-D-cultured HBMEC (Fig. 4C). To confirm these transcriptional changes, we next performed enzyme-linked immunosorbent assays (ELISAs) for IFN-β or the type III IFNs IFN-λ1 and IFN-λ2, which confirmed the enhanced induction of antiviral signals in 3-D-cultured HBMEC exposed to poly(I·C) (Fig. 4D; Fig. S3B and C). Interestingly, we also found that the enhanced capacity to mount antiviral signals was retained in 3-D^tryp^ cells, which also induced IFN-β, IFN-λ1, and IFN-λ2 in response to poly(I·C) stimulation more robustly than 2-D-cultured cells (Fig. 4D; Fig. S3B and C). Finally, we confirmed the differential capacity of 3-D-cultured cells to mount antiviral signals by RNAseq, which validated a more potent induction of IFNs and ISGs in HBMEC cultured in 3-D (Fig. 4E).

10.1128/mSphere.00206-17.3 FIG S3  (A) Induction of ISG60 as assessed by RT-qPCR in 2-D- or 3-D-cultured HBMEC exposed to 1 μg, 10 μg, or 20 μg of “floated” poly(I·C). (B and C) ELISAs for IFN-γ2 (B) and IFN-β (C) (with results expressed in picograms per milliliter) in 2-D- or 3-D-cultured HBMEC or in 3-D^tryp^ cells, exposed to 10 μg of poly(I·C). (D) RT-qPCR for IκBα and IL-8 in 2-D- or 3-D-cultured HBMEC exposed to LPS (500 ng/ml) or flagellin (100 ng/ml). In all panels, data are shown as means ± standard deviations and are normalized to mock-treated cells. (E) Induction of ISG56 as assessed by RT-qPCR in 2-D or 3-D^tryp^ HBMEC exposed to 10-μg poly(I·C) at various times posttrysinization. (F) Heat map of the expression of TLRs and RLRs and their associated adaptors (based on log[RPKM] values from RNASeq analyses) in 2-D- or 3-D-cultured HBMEC (gray denotes transcripts with no reads). (G) RT-qPCR for TLR3, RIG­I, and mitochondrial antiviral signaling protein (MAVS) in 2-D- or 3-D-cultured HBMEC. In panels A to E and G, data are shown as means ± standard deviations and are normalized to mock-treated cell results (A and B) (**, *P* < 0.01; ***, *P* < 0.001; ns, not significant). Download FIG S3, PDF file, 1.3 MB. Copyright © 2017 Bramley et al. 2017 Bramley et al. This content is distributed under the terms of the Creative Commons Attribution 4.0 International license.

We next determined whether the capacity to mount more potent antimicrobial signals in 3-D HBMEC was specific to antiviral pathways. Consistent with our findings using poly(I·C), we found that HBMEC cultured in 3-D induced antimicrobial signaling in response to LPS and flagellin more efficiently, as assessed by RT-qPCR for analysis of IκB alpha (IκBα) and interleukin-8 (IL-8) (Fig. S3D), by ELISA for analysis of IL-8 (Fig. 4F), and by RNAseq (Fig. 4G) in LPS-treated cells. Importantly, 3-D^tryp^ cells also retained this capacity (Fig. 4F; Fig. S3B to D) for up to 72 h postremoval (Fig. S3E). Collectively, these data show that the culturing of HBMEC in 3-D influences the capacity of these cells to respond to diverse microbial pathogen-associated molecular patterns (PAMPs), which is maintained for several days following removal of cells from beads and subsequent growth in 2-D. Importantly, we did not identify any changes in the expression of components of TLR3- or RLR-mediated antiviral signaling pathways between 2-D, 3-D, and primary cells by RNAseq or by RT-qPCR (Fig. S3F and G), suggesting that the differential induction observed did not result from alterations in pattern recognition receptor (PRR) expression.

### HBMEC cultured in 3-D resist infection by RNA viruses.

**FIG 5:**
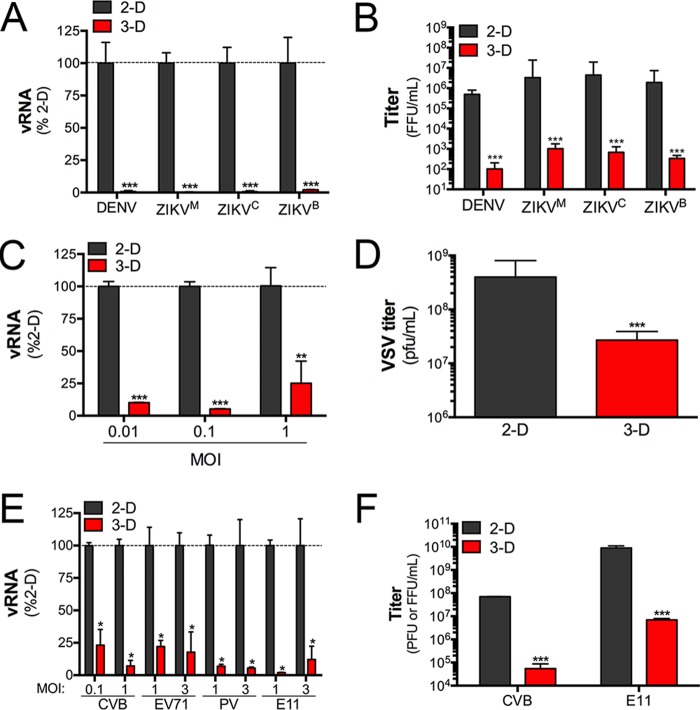
3-D-cultured HBMEC were resistant to ZIKV infection. (A) RT-qPCR analysis for viral RNA (vRNA) production in 2-D- or 3-D-cultured HBMEC infected with DENV or with three strains of ZIKV (MR766 [ZIKV^M^], FSS13025 [ZIKV^C^], or Paraiba/2015 [ZIKV^B^]). (B) Fluorescence focus assay for DENV or the indicated ZIKV strains in 2-D or 3-D cultures of HBMEC infected for 48 h. (C) RT-qPCR for analysis of vRNA production in 2-D- or 3-D-cultured HBMEC infected with the indicated multiplicity of infection (MOI) of VSV. (D) Plaque assays for VSV in 2-D or 3-D cultures of HBMEC infected with VSV (0.1 PFU/cell) for ~16 h. (E) RT-qPCR for analysis of vRNA production in 2-D- or 3-D-cultured HBMEC infected with the indicated enteroviruses (at the indicated MOIs) for 16 to 24 h. (F) Plaque (CVB) or fluorescence focus (E11) assays for CVB or E11 in 2-D or 3-D cultures of HBMEC. In all panels, data are representative of >3 independent STLVs and are shown as means ± standard deviations (in panels A to D and F, ** = *P* < 0.01 and *** = *P* < 0.001; in panel E, * = *P* < 0.001). Data are representative of cells isolated from at least three independent STLV cultures, with experiments performed in duplicate or triplicate for all cultures.

Because there were significant differences in the responses of 2-D- and 3-D-cultured HBMEC to synthetic ligands of key antiviral pathways, we next assessed whether there were differences in the susceptibility of 2-D- and 3-D-cultured HBMEC to RNA viruses, including members of the flavivirus, rhabdovirus, and enterovirus families. For flavivirus studies, we utilized three strains of Zika virus (ZIKV), one of African lineage (MR766; ZIKV^M^) and two of Asian lineages (including a strain from Cambodia [FSS13025; ZIKV^C^] and a strain from a febrile case in Brazil in 2015 [Paraiba/2015; ZIKV^B^]), as well as dengue virus (DENV, serotype 2). In all cases, we found that 3-D-grown HBMEC potently resisted infection by all strains of ZIKV and DENV as assessed by the low levels of production of viral RNA (vRNA) (Fig. 5A) and the significant (>10,000-fold) reduction in infectious viral titers (Fig. 5B). To confirm that this loss of infection did not result from alterations in receptor expression or other host factors that might dampen infection in a virus-specific manner, we next determined whether there were differences in the susceptibility of 2-D- and 3-D-cultured HBMEC to other RNA viruses using vesicular stomatitis virus (VSV) and members of the enterovirus family, including coxsackievirus B (CVB), enterovirus 71 (EV71), poliovirus (PV), and echovirus 11 (E11). We found that HBMEC cultured in 3-D potently resisted infection by all viruses, with significant differences in the production of vRNA and infectious viral particles (Fig. 5C to F).

### Resistance of 3-D-cultured HBMEC to RNA virus infection does not result from antiviral signaling.

**FIG 6:**
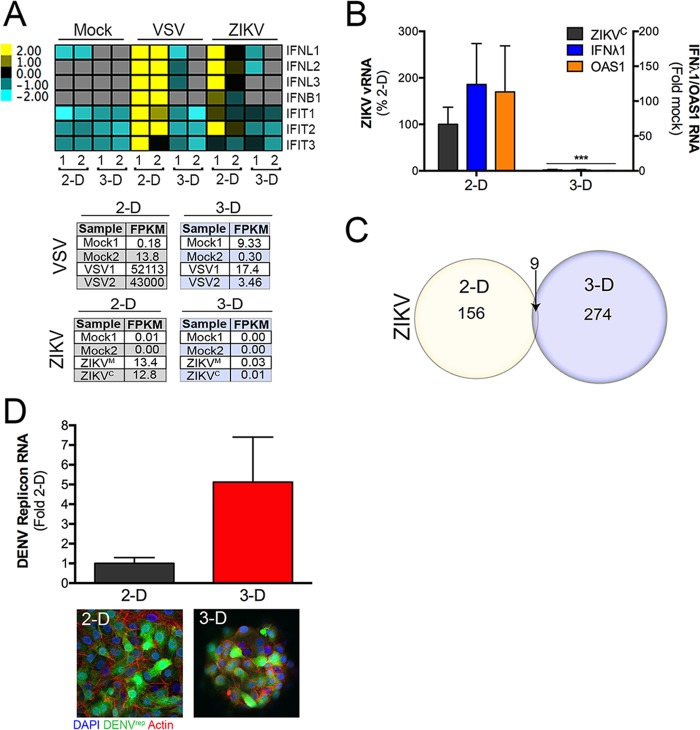
Restriction of ZIKV infection in 3-D-cultured HBMEC does not result from enhanced antiviral innate immune signaling. (A) Heat maps of transcripts associated with type I or III IFN antiviral signaling pathways in 2-D- or 3-D-cultured HBMEC that had been subjected to mock infection, VSV infection, or ZIKV infection for ~24 h (ZIKV^C^ [sample 1] or ZIKV^M^ [sample 2]), highlighting the lack of induction of potent antiviral pathways in virally infected 3-D cultures. The numbers at the bottom indicate independent replicates. The color intensity indicates the level of gene expression (yellow for upregulation and blue for downregulation), and gray indicates that no reads were detected for that transcript. The table at the bottom denotes FPKM values from mapped reads in mock-infected or VSV- or ZIKV-infected samples. (B) RT-qPCR for ZIKV^C^ infection (left *y* axis) or IFNλ1 or OAS1 expression (right *y* axis) in 2-D or 3-D cultures of HBMEC infected with ZIKV for 24 h. Data are shown as means and standard deviations and are normalized to 2-D (left *y* axis) or mock-infected (right *y* axis) controls. (C) Venn diagram showing the overlap (9 transcripts) of differentially expressed transcripts (as assessed by RNAseq) in 2-D or 3-D HBMEC cultures. (D) (Top) RT-qPCR for DENV vRNA in 2-D- or 3-D-cultured HBMEC stably propagating a GFP-fused DENV subgenomic replicon (DENV^rep^). (Bottom) Confocal micrographs from analyses of GFP and actin (red) in 2-D- or 3-D-cultured DENV^rep^ HBMEC. DAPI-stained nuclei are shown in blue. Data are shown as means ± standard deviations, are normalized as percent 2-D infection, and are representative of cells isolated from two independent STLV cultures.

Because our data showed that HBMEC grown in 3-D potently responded to synthetic ligands of antiviral innate immune pathways and resisted RNA virus infection, we next assessed whether the resistance of cells to infection resulted from innate immune signaling. First, we defined the global expression changes in 2-D and 3-D HBMEC infected with either VSV or ZIKV by RNAseq. We found that VSV and ZIKV infection of 2-D-cultured HBMEC induced IFN-λ1 to IFN-λ3, IFN-β, and ISGs (Fig. 6A). However, in contrast, there was minimal to no induction of any antiviral innate immune pathways in VSV- or ZIKV-infected HBMEC cultured in 3-D (Fig. 6A). Notably, supporting the data indicating a very low level of infection in 3-D-cultured cells infected with VSV or ZIKV, values representing numbers of fragments per kilobase of transcript per million mapped reads (FKTR) for both viral genomes were markedly lower in 3-D-cultured cells, which is indicative of the production of very low levels of viral RNA (Fig. 6A, bottom). We confirmed these RNAseq findings with RT-qPCR for ZIKV-infected 2-D and 3-D HBMEC cultures, which showed no detectable induction of antiviral signaling pathways in ZIKV-infected 3-D-cultured HBMEC (Fig. 6B).

Interestingly, differential expression analysis (performed using the DESeq2 package in R; 17) revealed that the expression levels of 156 transcripts were altered by ZIKV infection of 2-D-cultured HBMEC and that, despite the very low levels of viral RNA present, 274 transcripts were altered by ZIKV infection of 3-D-cultured HBMEC (Fig. 6C). GSEA revealed common antiviral and virus-induced pathways in ZIKV-infected 2-D-derived HBMEC (Fig. S4A). In contrast, 3-D cultures infected with ZIKV exhibited the induction of only IFN-γ signaling-associated components (FDR = 0.000) (Fig. S4B). However, despite significant transcriptional changes induced by ZIKV infection in 2-D- and 3-D-cultured HBMEC, only nine transcripts were shared between culture conditions (Fig. 6C). Collectively, these data suggest that the lack of susceptibility of 3-D-cultured HBMEC to viral infections does not result from the induction of antiviral innate immune signaling pathways.

10.1128/mSphere.00206-17.4 FIG S4  (A and B) Gene set enrichment analysis plots from 2-D (a)- or 3-D (b)-cultured HBMEC infected with ZIKV. Download FIG S4, PDF file, 2.5 MB. Copyright © 2017 Bramley et al. 2017 Bramley et al. This content is distributed under the terms of the Creative Commons Attribution 4.0 International license.

To determine whether the restriction of viral infection occurred at an early or late stage of the viral life cycle, we constructed HBMEC stably expressing a DENV subgenomic replicon fused to green fluorescent protein (GFP) (DENV^rep^) and established 3-D cultures using these cells. If the restriction of RNA virus infection in 3-D-cultured cells occurs at later stages of the viral life cycle, we expected that cells grown in 3-D would exhibit less viral RNA than 2-D cultures. In contrast, we found that HBMEC DENV^rep^ cells grown in 3-D exhibited no reductions in viral RNA levels and instead exhibited slightly enhanced vRNA levels (Fig. 6D, top). This was supported by the finding of equivalent levels of GFP fluorescence between 2-D- and 3-D-cultured HBMEC DENV^rep^ cells (Fig. 6D, bottom). Taken together, these data indicate that the lack of viral infection in 3-D-cultured cells does not result from the induction of innate immune signaling and that the inhibition occurs at an early stage of the viral life cycle, likely prior to the establishment of viral replication.

### The barrier properties of 3-D-cultured HBMEC restrict RNA virus infection.

**FIG 7:**
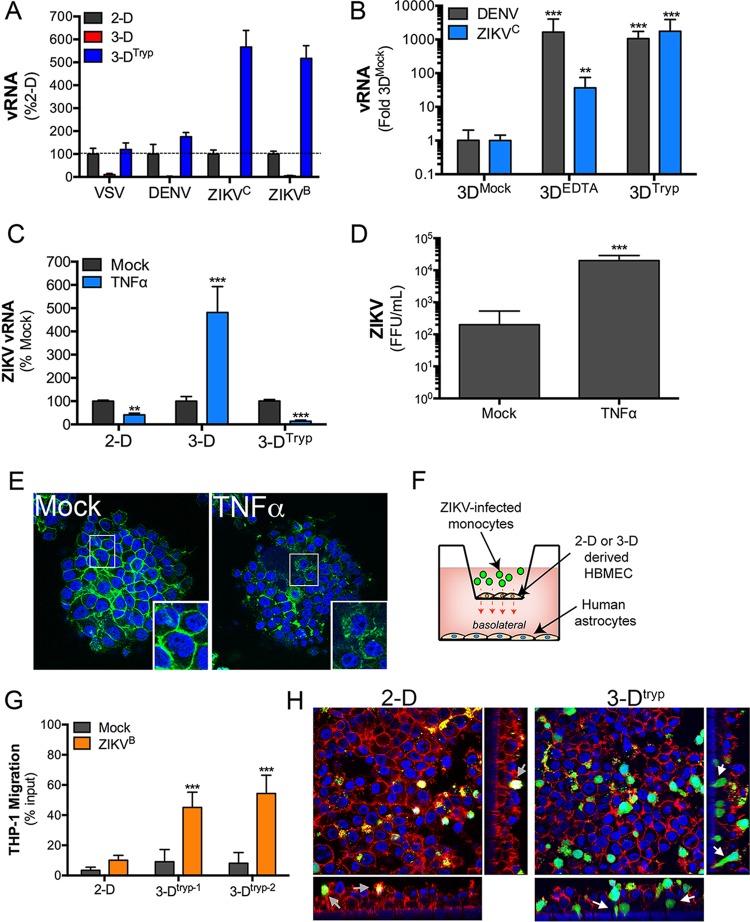
Apical junctions restrict RNA virus infection of 3-D-cultured HBMEC. (A) RT-qPCR for analysis of VSV, DENV, ZIKV^C^, or ZIKV^B^ infection viral RNA (vRNA) in 2-D, 3-D, or 3-D^tryp^ cultures of HBMEC. (B) RT-qPCR for DENV or ZIKV^C^ vRNA in 2-D, 3-D, or 3-D^tryp^ cultures of HBMEC treated with 10 mM EDTA for 1 h prior to infection. (C) RT-qPCR for ZIKV vRNA in 2-D, 3-D, or 3-D^tryp^ cultures of HBMEC that were treated with 100 ng/ml TNF-α for 24 h prior to infection with ZIKV^B^ for an additional 48 h (in the presence of TNF-α). (D) ZIKV infectious titers from mock- or TNF-α-treated 3-D-cultured HBMEC. (E) Confocal micrographs of mock- or TNF-α-treated 3-D cultures of HBMEC stained for actin (green). DAPI-stained nuclei are shown in blue. (F) Schematic for monocyte transmigration assay using ZIKV^B^-infected monocytes (shown in green), 2-D or 3-D^tryp^ HBMEC plated in the apical chamber, and primary human astrocytes plated in the basolateral compartment. (G) Quantification (performed in triplicate) of the percentages of ZIKV^B^-infected or control uninfected monocytes transmigrating from the apical to basolateral chambers across 2-D or two independent 3-D^tryp^ preparations. (H) Confocal micrographs of CFDA-labeled ZIKV^B^-infected THP-1 cells (green) 24 h following their addition to the apical chambers of Transwell inserts containing 2-D or 3-D^tryp^ cultures stained for actin (in red). DAPI-stained nuclei are shown in blue. THP-1 cells that remained in the apical surfaces of 2-D cells are shown with gray arrows, and THP-1 cells in the 3-D^tryp^ panels that were in the process of transmigrating the endothelial monolayer are shown with white arrows. Images are representative of cells isolated from two independent 3-D^tryp^ preparations in experiments performed in triplicate. Data in panels A to D and G are shown as means ± standard deviations and are normalized as a percentage of 2-D results (A), mock-treated cells (B and C), or percent input cells (G) (*, *P* < 0.05; **, *P* < 0.01; ***, *P* < 0.001).

We next determined whether 3-D-cultured cells removed from beads (3-D^tryp^) retained their capacity to resist viral infections, given in particular that these cells maintain some of the transcriptional and innate immune response profiles of cells cultured under exclusively 3-D conditions. We found that the susceptibility of 3-D^tryp^ cells to VSV, DENV, and ZIKV infection was fully restored in 3-D^tryp^ cells and that, in the case of ZIKV, infection was enhanced (Fig. 7A).

Given that the failure of RNA viruses to infect 3-D-cultured HBMEC was not attributable to enhanced antiviral innate immune signaling and that 3-D^tryp^ cells regained susceptibility to infection, we reasoned that the lack of infection in 3-D-cultured cells might result from the enhanced barrier properties of cells cultured only under 3-D conditions. The BBB forms a major physical barrier to the passage of viruses in the bloodstream into the CNS, a property that can be modulated as the result of the inflammatory cytokine signaling that accompanies viral infections (reviewed in reference 2). First, we determined whether treatment of 3-D-derived HBMEC with EDTA, which enhances paracellular permeability due to disruption of apical junctional complexes, would enhance RNA virus infections. Indeed, replication of both DENV and ZIKV^C^ was enhanced to levels more similar to those observed in 3-D^tryp^ cells when 3-D cultures were treated with EDTA (Fig. 7B). Next, we determined whether proinflammatory cytokines known to disrupt junctional complexes, such as TNF-α, and which are produced during flavivirus infections (31, 32), enhanced the susceptibility of 3-D cells to DENV and ZIKV infection. We found that ZIKV^C^ infection was enhanced in 3-D-cultured cells exposed to purified TNF-α compared to mock-treated controls (Fig. 7C and D). Interestingly, this same treatment inhibited ZIKV^C^ replication in both 2-D-cultured HBMEC and 3-D^tryp^ cells (Fig. 7C). TNF-α-treated HBMEC cultured in 3-D exhibited significant alterations in the integrity of their junctional networks, as assessed by actin localization (Fig. 7E).

In addition to the direct breakdown of the BBB by proinflammatory cytokines, viruses can access the CNS via passage across the BBB in virally infected immune cells (reviewed in reference 33). However, modeling this property has been challenging given the lack of appropriate *in vitro* cell systems that develop *in vivo*-like functions that recapitulate this process. Because HBMEC remain attached to beads during their 3-D culturing in the RWV bioreactor, assessing whether monocytes cross the microvascular endothelial barrier is not possible. Therefore, we used 3-D^tryp^ cells, which retain many of the phenotypic changes observed in 3-D-cultured cells attached to beads. Using these cells, we established a Transwell-based monocyte transmigration assay whereby 2-D or 3-D^tryp^ HBMEC were plated in the apical chamber of a Transwell insert and primary human astrocytes were plated in the basolateral chamber (schematic, Fig. 7F). After 3 days in culture, when both 2-D- and 3-D^tryp^-derived HBMEC exhibited maximal TER values (Fig. 3G), we added human monocyte THP-1 cells that had been infected with ZIKV^B^ for 5 days, or control uninfected THP-1 cells, to the apical chamber. To visualize THP-1 cells, we labeled them with carboxyfluorescein diacetate succinimidyl ester (CFDA SE), an amine-fixable and cell-permeative cell dye that fluoresces upon cleavage of its acetate group by intracellular proteases. Following incubation for ~24 h, we removed the medium from the basolateral chamber and counted the number of THP-1 cells that had migrated by automated cell counting. To ensure that these cells were THP-1 cells, we verified by microscopy that the isolated cells were positive for fluorescein. We observed a significant enhancement of the transmigration of ZIKV-infected THP-1 cells across 3-D^tryp^ cells compared to 2-D-cultured cells or control uninfected cells (Fig. 7G). These data were verified by confocal microscopy, whereby we observed the presence of CFDA-labeled THP-1 cells within the cell monolayer of 3-D^tryp^ cells but not 2-D cells, including several monocytes in the process of penetrating the Transwell membrane (Fig. 7H). In contrast, we found that THP-1 cells in 2-D-cultured HBMEC largely remained on the surface of the endothelium and exhibited minimal localization within the endothelial cell monolayer (Fig. 7H). Consistent with this, we observed an increase in ZIKV^B^ infection of primary astrocytes plated in the basolateral compartment of 3-D^tryp^ cells, indicating that the transmigration of infected THP-1 cells enhanced their infection (Fig. S5).

10.1128/mSphere.00206-17.5 FIG S5  ZIKV^B^ infection in primary astrocytes cultured in the basolateral compartment shown in schematic in 2-D or 3-D^tryp^ HBMEC incubated with ZIKV^B^-infected THP-1 cells in the apical chamber for ~24 h. Download FIG S5, PDF file, 0.2 MB. Copyright © 2017 Bramley et al. 2017 Bramley et al. This content is distributed under the terms of the Creative Commons Attribution 4.0 International license.

## DISCUSSION

Neurotropic viral infections represent a major burden to human health. Thus, developing models and tools by which to study the mechanisms by which RNA viruses access the CNS is essential in order to rapidly and easily test antiviral therapeutics. The lack of appropriate *in vitro* human-based BBB models is an impediment for the testing of both noninfectious disease- and infectious disease-based therapeutics, which requires that the system reflect the unique properties of the BBB *in vivo* and provide sufficient cell numbers for high-throughput screening (HTS) approaches. Here, we describe a 3-D-based model of the human BBB that yields large numbers of cells that maintain many essential characteristics of the BBB *in vivo*, including their transcriptional profile, barrier properties, antimicrobial signaling properties, and resistance to viral infections. Our data show that this model can be used to profile the mechanisms by which RNA viruses access the CNS, which could include proinflammatory cytokine-mediated dysfunction of the BBB microvascular endothelium barrier function and monocyte-mediated transmigration. These findings could inform our understanding of the processes by which viruses might access the CNS and provide a human cell-based platform that can be used to test the effectiveness of antiviral therapeutics at restricting these events.

The system that we describe here has several advantages, including the ability to generate large numbers of cells with more *in vivo*-like phenotypes and exposure to physiologically relevant levels of shear throughout their culture period. Presumably, these changes are all induced by the exposure of HBMEC to shear forces induced by culturing in the RWV bioreactor. This conjecture is at least in part supported by our findings showing that many of the transcripts differentially expressed in 3-D-cultured HBMEC are also differentially expressed when another BBB microvascular endothelial cell line (hCMEC/D3) is cultured in 3-D. Given that the exposure of BBB endothelial cells to shear forces is known to induce changes in gene expression (12, 13), these data suggest that the shear forces induced by the culturing of HBMEC in 3-D significantly alter gene expression. To our knowledge, there are no existing whole-genome transcriptional profiling data sets from chip-based culturing of BBB endothelial cells to compare our RWV bioreactor-based culturing data sets to; thus, it is unclear whether other factors associated with bioreactor-based culturing (i.e., membrane curvature associated with bead-based culturing, differences in mass transfer, etc.) impact the changes we observe. In addition, differences in viral susceptibility and/or innate immune signaling differences in chip-based models have not been explored to our knowledge. Although the system that we describe does have several advantages, including the ability to generate large cell numbers that can be used for parallel experiments from a single-cell seeding and low fluid-to-cell ratios, limitations of the system include the need to culture cells for extended periods of time (~21 days) and the requirement to establish the RWV bioreactor system.

The most striking differences that we observed between 3-D-cultured cells and those that had been removed from beads postculturing (3-D^tryp^) related to the dramatic differences in susceptibility of these different cell cultures to viral infections. These findings are particularly striking given that 3-D^tryp^ cells retain their ability to mount more-robust antimicrobial innate immune signals than 2-D-cultured cells for up to 72 h post-bead removal. These data would suggest that, at least in the culture system that we describe, the physical barrier presented by BBB microvascular endothelial cells is more significant in restricting neurotropic viral infections than their ability to mount antiviral signaling. In mouse models of West Nile virus (WNV) BBB infections, this would also seem to be the case given that the antiviral effects of IFN-λ are directly linked to the ability to strengthen the barrier function of the endothelium rather than to the ability to induce antiviral ISGs (34). Interestingly, we found that 3-D (both “on bead” and 3-D^tryp^)-cultured HBMEC potently induced type III IFNs in response to synthetic ligand stimulation of TLRs or RLRs and to viral infections to levels greater than those observed for type I IFNs. However, the upstream and downstream basis for these differences remains unknown. Collectively, our data suggest that the “on bead” 3-D HBMEC culture system that we describe would be ideal to model the physical and immunological barrier that might be presented by the BBB endothelium *in vivo*, whereas the “off bead” cells (3-D^tryp^) might be appropriate to model the immunological barrier properties without fully recapitulating the barrier properties.

Breakdown of BBB TJ *in vivo* by proinflammatory cytokines could expose the underlying brain tissue to viral particles via paracellular transport across the monolayer and/or enhance viral infection in the microvascular endothelium itself. Our data show that in the 3-D model of microvascular endothelial cells that we describe, TNF-α exposure significantly enhanced ZIKV infection of the endothelium itself. Although the full range of host cell molecules that facilitate ZIKV binding and entry is not understood, phosphatidylserine receptors that are members of the TIM and TAM families may function in ZIKV infection (35) as these receptors also play roles in DENV attachment and entry (36). Notably, these receptors do not appear to be associated with ZIKV-induced disease in small-animal models (37). On the basis of our transcriptional profiling data, there were no changes in the expression levels seen with members of the TIM or TAM families induced by the culturing of HBMEC under 3-D conditions. However, it is unknown whether members of this family exhibit any asymmetric localization in fully polarized cells, although our data suggest that these receptors, or other receptors that play roles in ZIKV infection, might exhibit a more basolateral distribution in polarized BBB microvascular endothelial cells, which would explain the enhancement of ZIKV infection by TNF-α treatment.

Here we report the development of a 3-D model system of the human BBB that can be applied to the study of antiviral signaling and viral entry into the CNS. Using this system, we show that ZIKV fails to efficiently replicate in 3-D-cultured BBB endothelial cells but can bypass this barrier via cytokine-mediated disruption of endothelial junctions or in infected monocytes. Thus, we have established a platform by which to model a variety of aspects of viral infections of the BBB that could be applied to the screening and development of antiviral therapeutics.

## MATERIALS AND METHODS

### Cells and viruses.

HBMEC (grown under either 2-D or 3-D conditions) were cultured in RPMI 1640 supplemented with 10% fetal bovine serum (FBS), 10% NuSerum, nonessential amino acids (NEAA), minimum essential medium (MEM) vitamins, sodium pyruvate, antibiotics, and endothelial cell growth supplement, as previously described (9). Primary HBMEC were isolated and cultured as previously described (9). *Aedes albopictus* midgut C6/36 cells were provided by Jared Evans (Johns Hopkins Applied Physics Laboratory) and were maintained in Dulbecco’s modified Eagle’s medium (DMEM) supplemented with 10% FBS and antibiotics at 28°C in a 5% CO_2_ air atmosphere, and Vero cells (provided by Fred Homa [University of Pittsburgh]) were maintained in DMEM supplemented with 5% FBS and antibiotics. THP-1 cells (obtained from InvivoGen) were propagated in RPMI 1640 supplemented with 10% FBS and antibiotics. Primary normal human astrocytes (NHA) were purchased from Lonza and were maintained in astrocyte basal growth medium (ABM) supplemented with the necessary BulletKit to generate complete astrocyte growth medium (AGM) (Lonza). Primary human pericytes were purchased from Sciencell and were maintained in complete pericyte medium (Sciencell). Production of DENV^rep^ HBMEC was performed as previously described (38) utilizing replicon constructs provided by Theodore Pierson (NIH/NIAID).

DENV-2 16681 and ZIKV MR766 (Ugandan origin, obtained from the ATCC), FSS13025 (Cambodian origin, obtained from Robert Tesh, University of Texas Medical Branch), and Paraiba/2015 (provided by David Watkins, University of Miami) were propagated in C6/36 or Vero cells, as previously described (39). DENV and ZIKV titers were determined by fluorescent focus assay, as previously described (40), using recombinant anti-double-stranded RNA monoclonal antibody (provided by Abraham Brass, University of Massachusetts). Propagation and titration of VSV (Indiana strain) and of CVB, PV, EV71, and E11 have been described previously (41). Experiments measuring productive infection were performed with 0.1 to 3 PFU/cell or 0.1 to 3 focus-forming units (FFU)/cell for 24 to 48 h, as stated in the figure legends. Infection was determined by RT-qPCR and/or plaque assays (VSV, CVB) or fluorescent focus assays (DENV, ZIKV, E11) as stated in the figure legends. The methods used for infection of THP-1 cells with ZIKV are described below (under "Monocyte transmigration assays"). For infections of cells grown in 3-D, beads were removed from the STLV and infected immediately in 24-well plates for the duration of the infection period (24 to 48 h). For calculations of cell number, beads were removed from the STLV, washed with phosphate-buffered saline (PBS), and incubated with 0.05% trypsin–EDTA to remove cells from beads and cells were counted using a Bio-Rad TC20 automated cell counter. In all cases, infections were performed with equivalent numbers of 2-D, 3-D, and 3-D^tryp^ cells.

### Rotating wall vessel bioreactor 3-D cultures.

HBMEC propagated as described above were harvested in 0.05% trypsin–EDTA and incubated with ~300 mg collagen-coated Cytodex-3 beads (Sigma) at 6 × 10^6^ cells/300 mg beads. After a brief (~30-to-60-min) static incubation at 37°C, the bead/cell slurry was added to autoclavable 55-ml slow-turning lateral vessels (STLVs) and was attached to a rotating base (Synthecon) at 19 to 21 rpm to maintain the cells in suspension for the duration of the culture period (21 days). Cell culture medium was replenished daily for the entirety of the 21-day culture period. For removal of HBMEC from cytodex beads (3-D^tryp^), 3-D-cultured cells were removed from the STLV and were rinsed with phosphate-buffered saline before being incubated with 0.05% trypsin–EDTA for ~45 min at 37°C with frequent rotation. Following trypsin incubation, the cell/bead slurry was passed through a 100-μm-pore-size cell strainer to remove beads. Isolated cells were then collected by centrifugation (1,200 rpm) and enumerated using a Bio-Rad TC20 automated cell counter. Isolated cells (3-D^tryp^) were then plated in 8-well chamber slides for imaging (described below), in 24-well plates for infections, or in Transwell inserts for measurements of TER (described below).

### PAMP stimulation.

Immune stimulation of 2-D and 3-D cultures was conducted using polyriboinosinic:polyribocytidylic acid [poly(I·C); InvivoGen], lipopolysaccharide (LPS) (Sigma), or flagellin (InvivoGen). Poly(I·C) (1 to 20 μg/ml) was administered directly to cell culture medium or transfected (1 μg) using Lipofectamine 2000. LPS (500 ng/ml) and flagellin (100 ng/ml) were added directly to the cell culture media. Treated cells were incubated for ~24 h prior to RNA and/or supernatant collection for downstream analyses such as RT-qPCR, RNAseq, and/or ELISA.

### Enzyme-linked immunosorbent assays.

ELISAs for analysis of human IL-8, IFN-β, IFN-λ1, and IFN-λ2 (all from R&D Systems) were performed according to the manufacturer’s protocol.

### Microscopy.

For imaging studies, cells or beads were fixed in 4% paraformaldehyde (PFA) or 100% ice-cold methanol followed by permeabilization in 0.25% Triton X-100, washed in PBS, incubated with primary antibody for 1 h, washed in PBS, and then incubated with Alexa Fluor-conjugated secondary antibodies for 30 min. Alexa Fluor 594-conjugated phalloidin and Alexa Fluor 488-conjugated phalloidin were used for actin localization and were purchased from Invitrogen. Mouse anti-Na/K ATPase was purchased from Millipore, mouse anti-VE-cadherin was purchased from R&D Systems, and rabbit anti-ZO-3 antibody and mouse anti-claudin-5 antibody were purchased from Invitrogen. Mouse anti-glial fibrillary acidic protein (anti-GFAP) antibody was purchased from BD Biosciences. Slides/beads were mounted with Vectashield (Vector Laboratories) containing 4′,6-diamidino-2-phenylindole (DAPI). Images were captured on an Olympus FV1000 confocal microscope or an IX83 inverted fluorescence microscope and were analyzed using ImageJ. For scanning electron microscopy (SEM), beads were fixed, processed, and imaged as described previously (42).

### RNA isolation and RT-qPCR.

Cellular RNA was isolated using a GenElute Total RNA MiniPrep kit (Sigma). Following isolation and treatment with DNase (Sigma), RNA was reverse transcribed using an iScript cDNA synthesis kit (Bio-Rad) containing 1 μg of sample RNA per reaction. RT-qPCR was conducted using IQ SYBR green SuperMix (Bio-Rad) in a Bio-Rad CFX96 Touch real-time PCR detection system as well as an Applied Biosystems StepOne Plus real-time PCR machine. A modified threshold cycle (Δ*C*_*T*_) method was used to calculate gene expression using human actin for normalization. Primer sequences for actin, ZIKV, IFN-β, IFN-λ, and ISGs have been previously published (43). Additional primer sequences can be found in Table S2 in the supplemental material.

10.1128/mSphere.00206-17.7 TABLE S2  List of qPCR primers used in the study. Download TABLE S2, PDF file, 0.02 MB. Copyright © 2017 Bramley et al. 2017 Bramley et al. This content is distributed under the terms of the Creative Commons Attribution 4.0 International license.

### RNAseq.

Total RNA was extracted as described above, and RNAseq was performed as described previously (42, 43). Briefly, libraries were prepared with a NEB Ultra Library preparation kit and library quality was determined using the Qubit assay and an Agilent 2100 Bioanalyzer. Sequencing was performed with an Illumina HiSeq 2500 system in rapid-run mode on one flow cell (two lanes), and CLC Genomics workbench 8 or 9 (Qiagen) was used to process, normalize, and map sequence data to the human reference genome (hg19). For the calculation of viral FPKM values, sequences were mapped to the appropriate viral genome using CLC Genomics workbench 9.0. Differentially expressed genes were identified using DESeq2 (17) with the significance cutoff value stated in the figure legends. Hierarchical clustering was performed using Cluster 3.0/Java TreeView. Heat maps were generated using MeViewer software based upon log(RPKM) values. GSEA v2. 2.0 (18) was used for the described enrichment analyses, which were deemed significantly enriched if the false-discovery rate (FDR) was <0.01. Files associated with RNAseq studies have been deposited into the BioProject database.

### TER measurements, TNF-α treatments, and monocyte transmigration assays.

For TER studies, HBMEC were removed from cytodex beads (to generate 3-D^tryp^ cells) as described above. Isolated cells (or 2-D-cultured control cells) were then seeded in the apical chamber of 6.5-mm-diameter or 12-mm-diameter 0.4-μm-pore-size collagen-coated Transwell inserts (T-col) (Corning) at a density of 5 × 10^4^ cells/well (6.5 mm) or 5 × 10^5^ cells/well (12 mm) in complete HBMEC medium. In the basolateral chamber, primary human astrocytes (1 × 10^5^ cells/well) were plated in normal growth medium (NGM). Alternatively, primary pericytes (5 × 10^4^ cells/well) were plated on the basolateral side of Transwell inserts in pericyte complete medium. TER values were measured at the indicated times using an epithelial volt/ohm meter (EVOM) (World Precision Instruments, Inc.).

For TNF-α studies, the indicated cells were treated with 100 ng/ml TNF-α (Sigma) for 24 h prior to the initiation of infection and were then infected with ZIKV in the presence of TNF-α for the duration of the infection (24 to 48 h).

THP-1 monocytes were infected with ZIKV for 5 days prior to transmigration assays. For these assays, 2-D or 3-D^tryp^ cells were added to the apical chamber of 6.5-mm-diameter 3.0-μm-pore-size Transwell inserts (T-clear) (Corning) at a density of 5 × 10^4^ cells/well in complete HBMEC medium. In the basolateral chamber, primary human astrocytes (1 × 10^5^ cells/well) were plated in NGM. ZIKV-infected THP-1 cells (5 × 10^4^) were labeled with CFDA according to the manufacturer’s protocol (Invitrogen) and added to the apical chamber and incubated for 24 h, at which time the medium in the basolateral chamber was collected and the transmigrated cells were enumerated using a Bio-Rad TC20 automated cell counter. Confirmation of the fluorescence of transcytosed CFDA-labeled THP-1 cells was confirmed by fluorescence microscopy using an IX50 inverted Olympus microscope and comparing the numbers of cells present (by bright-field microscopy) that were positive for fluorescein. For imaging, Transwells were fixed and stained as described above.

### Statistics.

All statistical analysis was performed using GraphPad Prism. Experiments were performed at least three times using at least two or three independent STLVs as indicated in the figure legends or as detailed for a minimum of at least six to nine technical replicates and two or three biological replicates. Data are presented as means ± standard deviations. Student’s *t* test or one-way analysis of variance (ANOVA) with Bonferroni’s correction was used for *post hoc* multiple comparisons to determine statistical significance, where appropriate. Specific *P* values are detailed in the figure legends.

### Accession number(s).

Files associated with RNAseq studies have been deposited in the BioProject database (BioProject ID PRJNA344703).
