# Supplementary figures and images for: A Three-Dimensional Cell Culture System To Model RNA Virus Infections at the Blood-Brain Barrier
Source: mSphere. 2017 Jun 21;2(3):e00206-17. doi: 10.1128/mSphere.00206-17 (PMC5480033; doi:10.1128/mSphere.00206-17)

Supplemental Figure 1

A

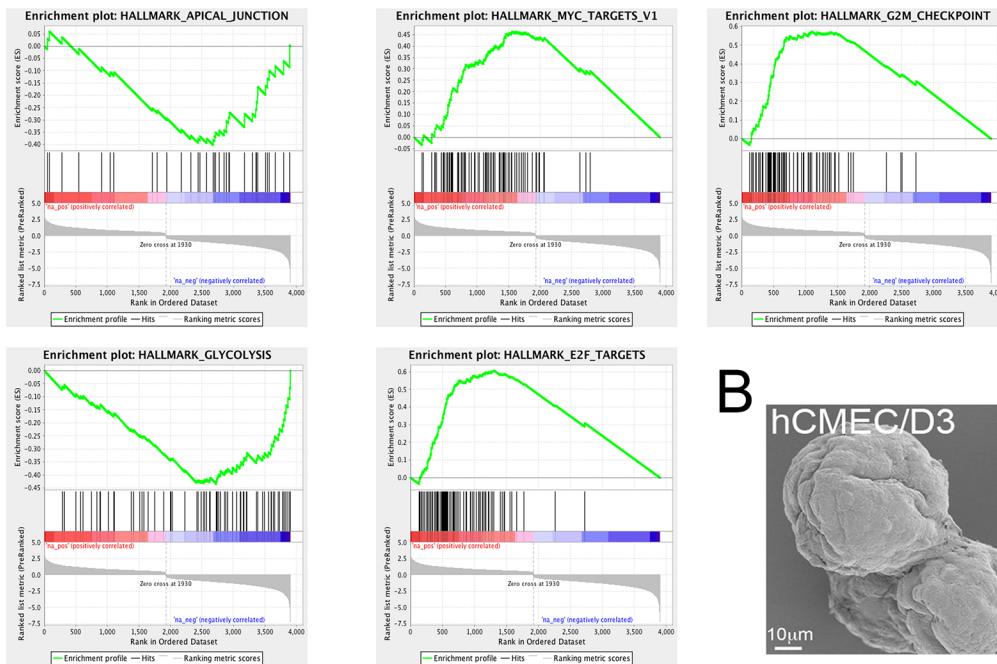

C

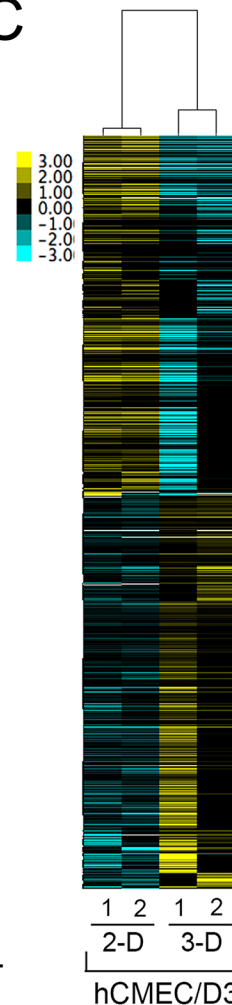

B

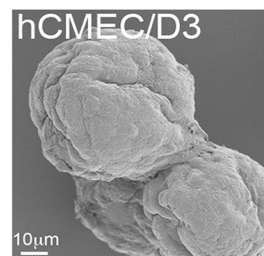

D

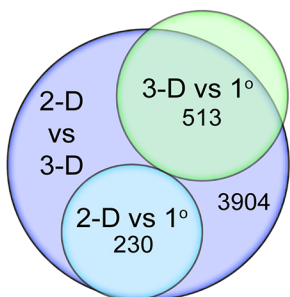

E

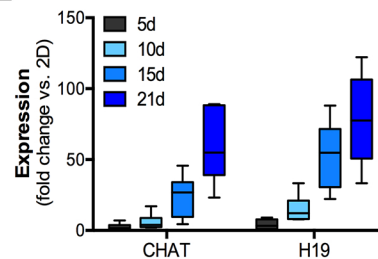

F

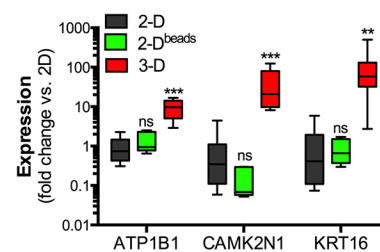

G

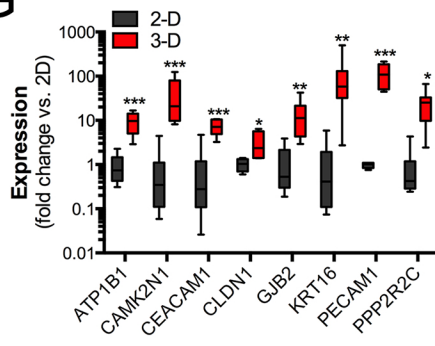

Supplement: FIG S1 [file sph003172306sf1.pdf]

Supplemental Figure 2

A

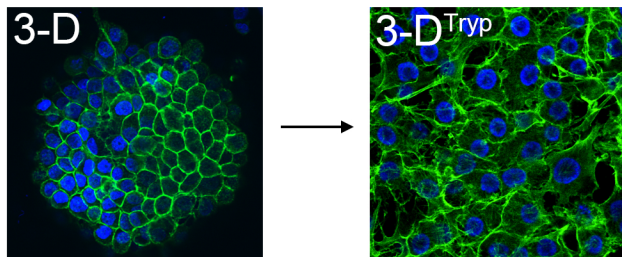

B

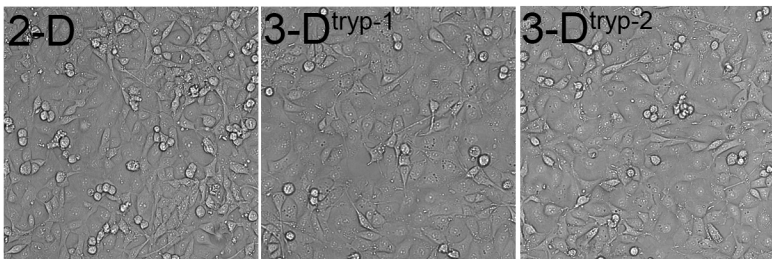

C

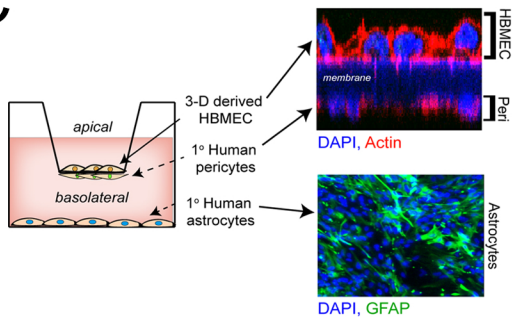

Supplement: FIG S2 [file sph003172306sf2.pdf]

A

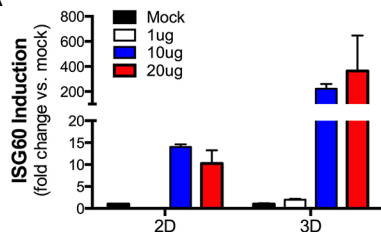

B

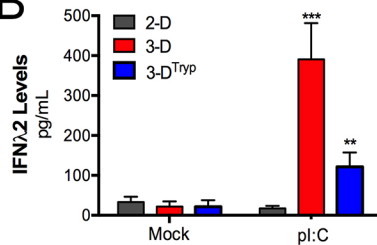

G

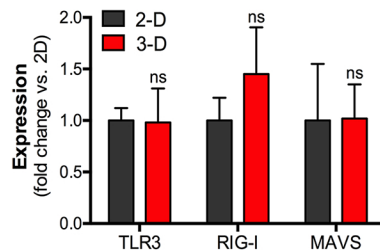

C

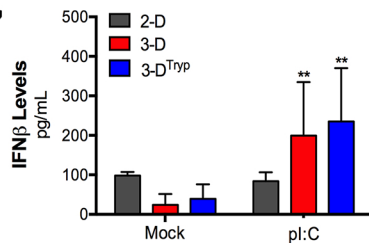

D

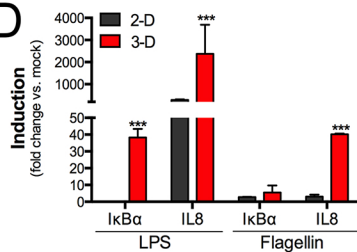

E

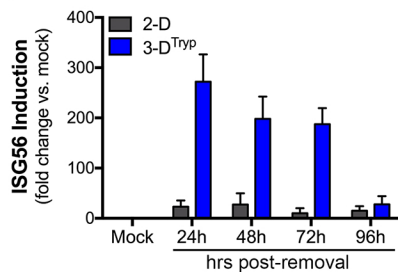

F

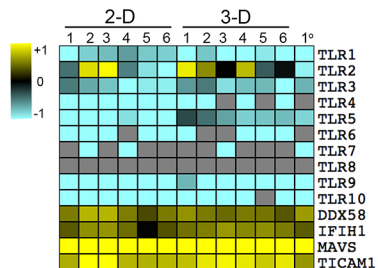

Supplement: FIG S3 [file sph003172306sf3.pdf]

Supplemental Figure 4

A

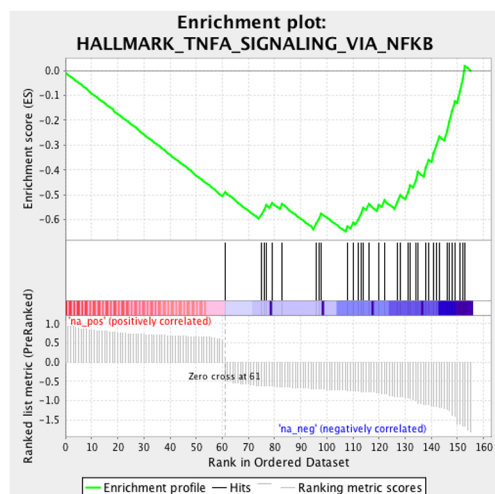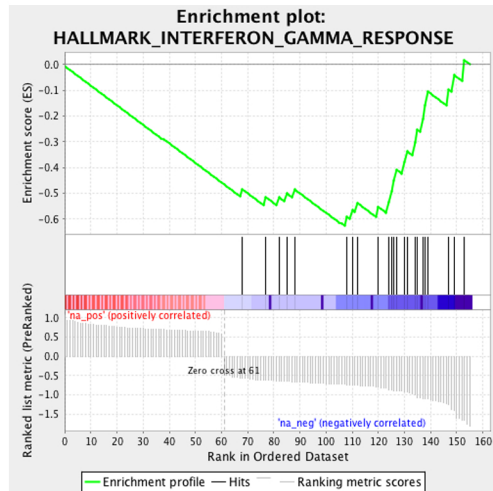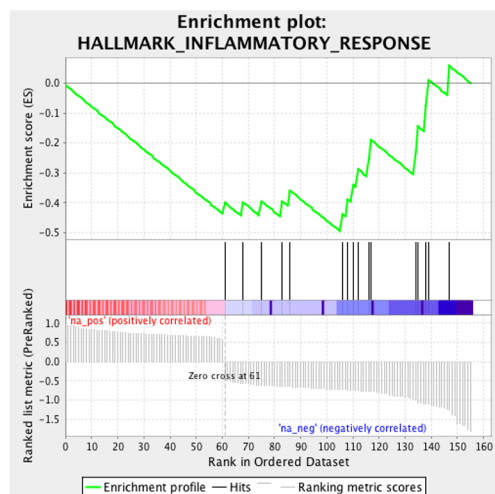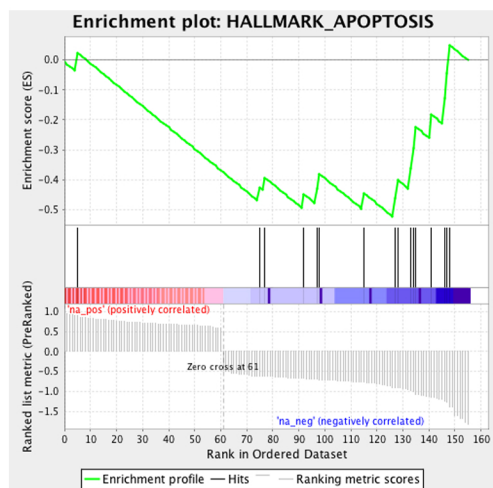

B

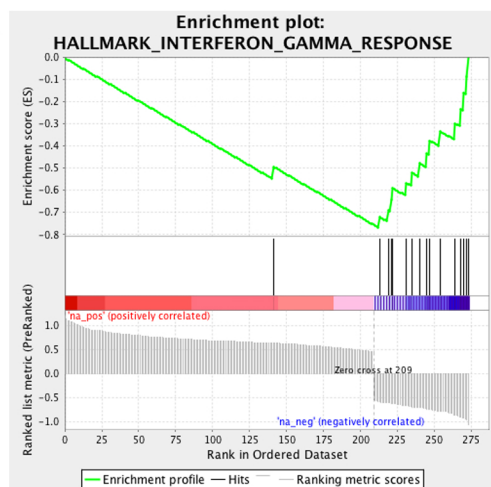

Supplement: FIG S4 [file sph003172306sf4.pdf]

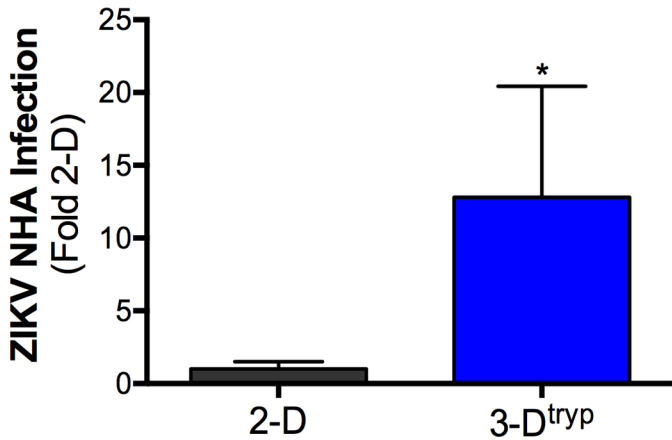

Supplement: FIG S5 [file sph003172306sf5.pdf]
