# Supplementary material for: A Three-Dimensional Cell Culture System To Model RNA Virus Infections at the Blood-Brain Barrier
Source: mSphere. 2017 Jun 21;2(3):e00206-17. doi: 10.1128/mSphere.00206-17 (PMC5480033; doi:10.1128/mSphere.00206-17)
Supplement: TABLE S2 [file sph003172306st7.pdf]

Gene	Forward (5'-3')	Reverse (5'-3')
ATP1B1	CCAGGATTAACACAGATTCC	GTTCAGTACATATGCCTCATAG
CAMK2N1	ATTCTGTATGTTGCACCTTG	TTGAGACACAGGAACAATTC
CEACAM1	CCACCTAACAAGATGAATGAAG	GAATCTCCTAGTGATGAGGG
CLDN1	TTGGCATGAAGTGTATGAAG	ACCTGCAAGAAGAAATATCG
CLDN5	TCTTTACTCCATCGGCAG	TTTTGGAGAGAGTTCAAACC
GJB2	AGGATATCGGCATTTGTTTC	GAAAGTTGGCAAAAAGAACC
PECAM1	AGATACTCTAGAACGGAAGG	CAGAGGTCTTGAAATACAGG
PPP2R2C	AACCAGAGAGTAAAAATGCG	GATAGTTTTATCGTTGGTGGAC
TJP3	CAAGATCATCAAACTAGACACC	GGATGAAGAAGACCACAATG

Supplemental Table 2. List of qPCR primers used in the study**.**
